# Supplementary figures and images for: Structural and biophysical analysis of a Haemophilus influenzae tripartite ATP-independent periplasmic (TRAP) transporter
Source: eLife. 2024 Feb 13;12:RP92307. doi: 10.7554/eLife.92307 (PMC10942642; doi:10.7554/eLife.92307)

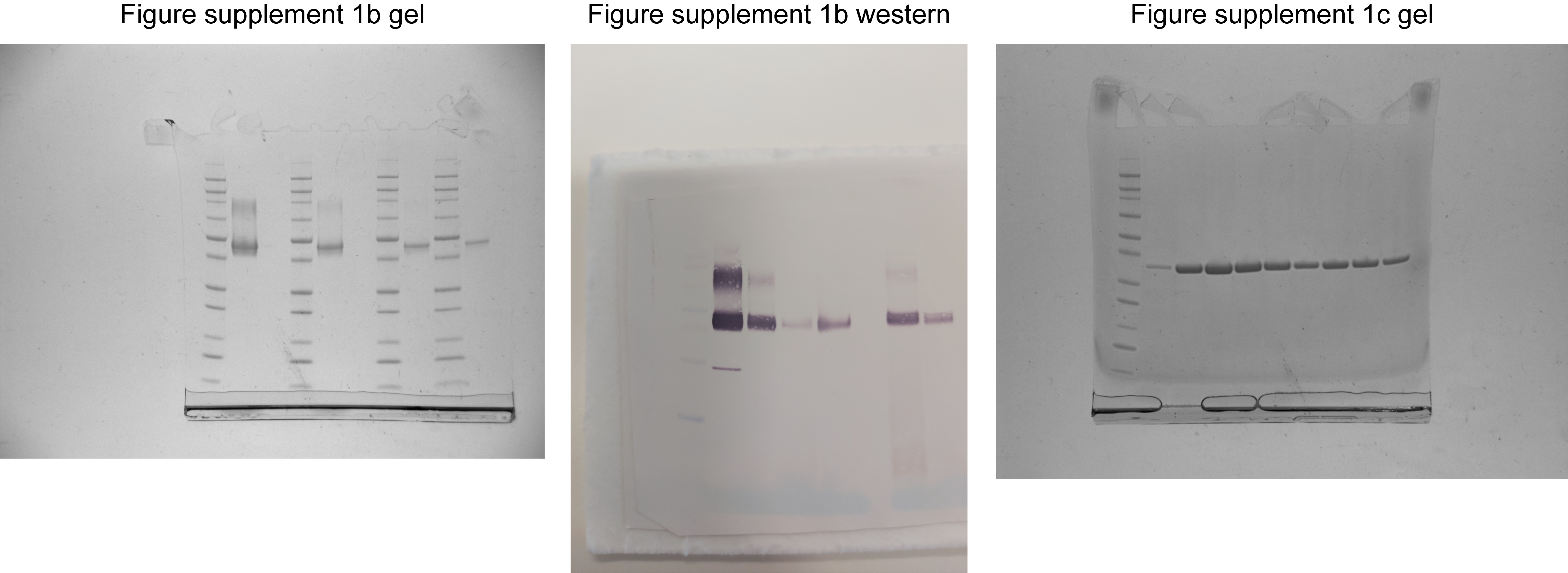

Supplement: Figure 2—figure supplement 1—source data 1. [file elife-92307-fig2-figsupp1-data1.zip › Figure 2-figure supplement 1-source data 1.tif]

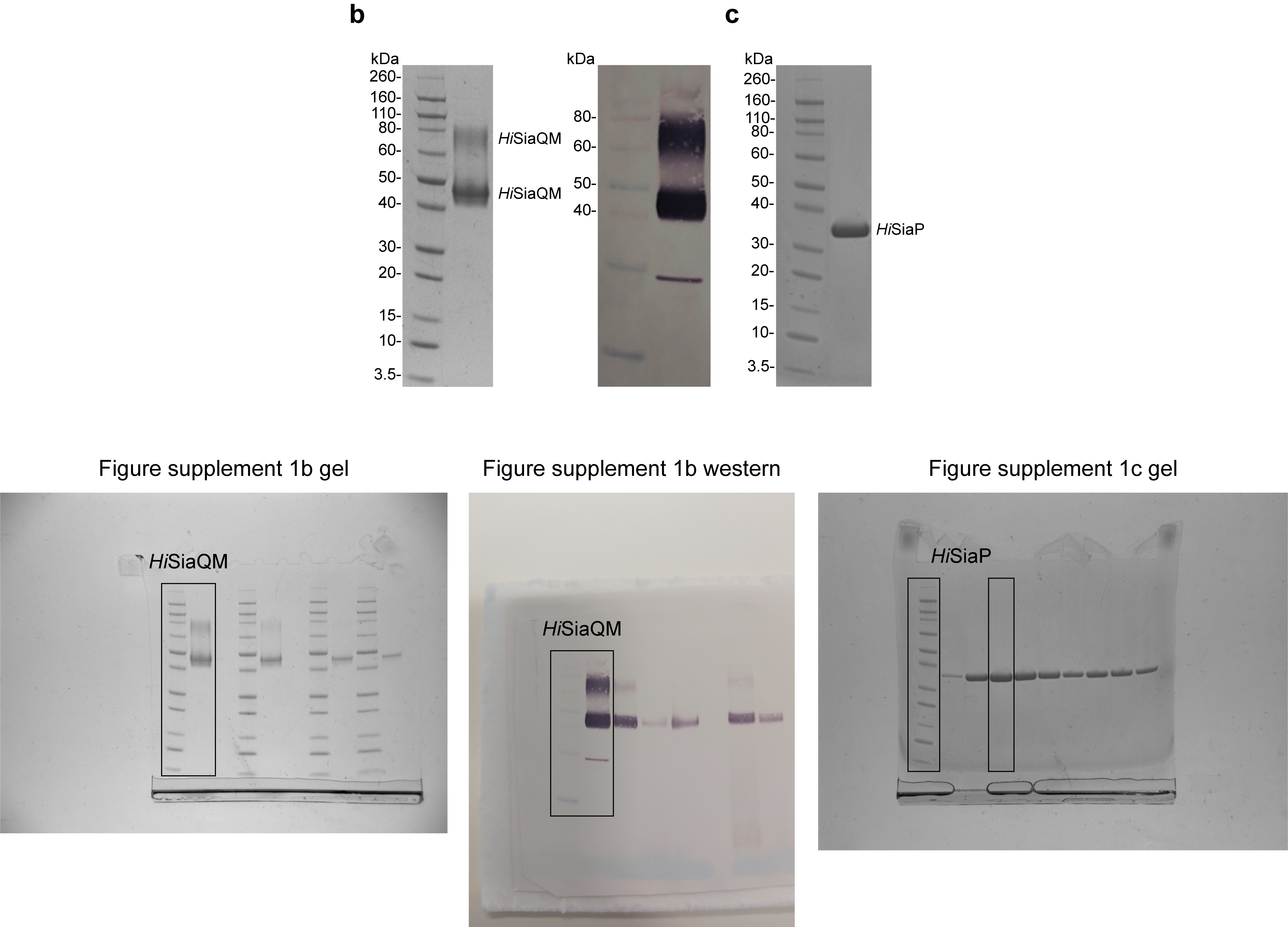

Supplement: Figure 2—figure supplement 1—source data 2. [file elife-92307-fig2-figsupp1-data2.zip › Figure 2-figure supplement 1-source data 2.tif]
