# Supplementary material for: Structural and biophysical analysis of a Haemophilus influenzae tripartite ATP-independent periplasmic (TRAP) transporter
Source: eLife. 2024 Feb 13;12:RP92307. doi: 10.7554/eLife.92307 (PMC10942642; doi:10.7554/eLife.92307)
Supplement: Supplementary file 1. [file elife-92307-supp1.docx]

|  | **Data collection** | |
| --- | --- | --- |
| Instrument | Titan Krios-beta at 300 kV | |
| Detector | K3 BioQuantum | |
| Camera | EF-CCD | |
| Mode | Counted super resolution | |
| Magnification | 130,000x | |
| Pixel size (Å/px) | 0.6645 | |
| C2 aperture (µm) | 50 | |
| Slit width (eV) | 20 | |
| Spot size | 5 | |
| Spherical aberration (mm) | 2.7 | |
| Frames | 163 | |
| Exposure time (s) | 2.18 | |
| Dose | 73.1 | |
| Fractions | 40 | |
| De-focus range | -2.0, -1.8, -1.6, -1.4, -1.2, -1.0, -0.8, -0.6, -0.4 | |
| Shots per hole | 10 | |
| Images collected | 14,281 | |
| Electrons/px/s | 14.798 | |
| Objective aperture | No | |
|  | ***Hi*SiaQM (parallel dimer)** | ***Hi*SiaQM (antiparallel dimer)** |
| **Processing** |  |  |
| Initial particle images (no.) | 2,950,415 | 2,950,415 |
| Final particle images (no.) | 220,810 | 225,044 |
| **Refinement** |  |  |
| Map resolution (Å) | 3.36 | 2.99 |
| FSC threshold | 0.143 | 0.143 |
| Model composition |  |  |
| Non-hydrogen atoms | 9521 | 9644 |
| Protein residues | 1224 | 1230 |
| Ligands | 4 | 8 |
| R.m.s. deviations |  |  |
| Bond lengths (Å) | 0.003 | 0.003 |
| Outliers | 0 | 0 |
| Bond angles (°) | 0.510 | 0.524 |
| Outliers | 1 | 0 |
| **Validation** |  |  |
| MolProbity score | 1.09 | 1.40 |
| Clash score | 3.02 | 5.25 |
| Rotamer outliers (%) | 0.29 | 0.88 |
| EMRinger score | 1.73 | 3.55 |
| Ramachandran plot |  |  |
| Favoured (%) | 98.2 | 97.39 |
| Allowed (%) | 1.8 | 2.61 |
| Outliers (%) | 0 | 0 |
| CC_volume_ | 0.80 (0.86*) | 0.86 |
| CC_mask_ | 0.80 (0.86*) | 0.87 |
| CC_peaks_ | 0.60 (0.77*) | 0.70 |
| PDB ID | 8THI | 8THJ |

*values from model fit to consensus parallel dimer map
