# Supplementary material for: Structural and biophysical analysis of a Haemophilus influenzae tripartite ATP-independent periplasmic (TRAP) transporter
Source: eLife. 2024 Feb 13;12:RP92307. doi: 10.7554/eLife.92307 (PMC10942642; doi:10.7554/eLife.92307)
Supplement: Supplementary file 2. [file elife-92307-supp2.docx]

| **Protein** | **Protein sequence** |
| --- | --- |
| *Hi*SiaQM  <https://www.uniprot.org/uniprot/P44543>  * X denotes the N-terminal purification tag | MGGSHHHHHHGMASMTGGQQMGRDLYDDDDKDRWGSELEMKYINKLEEWLGGALFIAIFGILIAQILSRQVFHSPLIWSEELAKLLFVYVGMLGISVAVRKQEHVFIDFLTNLMPEKIRKFTNTFVQLLVFICIFLFIHFGIRTFNGASFPIDALGGISEKWIFAALPVVAILMMFRFIQAQTLNFKTGKSYLPATFFIISAVILFAILFFAPDWFKVLRISNYIKLGSSSVYVALLVWLIIMFIGVPVGWSLFIATLLYFSMTRWNVVNAATEKLVYSLDSFPLLAVPFYILTGILMNTGGITERIFNFAKALLGHYTGGMGHVNIGASLLFSGMSGSALADAGGLGQLEIKAMRDAGYDDDICGGITAASCIIGPLVPPSIAMIIYGVIANESIAKLFIAGFIPGVLITLALMAMNYRIAKKRGYPRTPKATREQLCSSFKQSFWAILTPLLIIGGIFSGLFSPTESAIVAAAYSVIIGKFVYKELTLKSLFNSCIEAMAITGVVALMIMTVTFFGDMIAREQVAMRVADVFVAVADSPLTVLIMINALLLFLGMFIDALALQFLVLPMLIPIAMQFNIDLIFFGVMTTLNMMVGILTPPMGMALFVVARVGNMSVSTVTKGVLPFLIPVFVTLVLITIFPQIITFVPNLLIP |
| *Hi*SiaP  <https://www.uniprot.org/uniprot/P44542>  * X denotes the periplasmic signal peptide | MMKLTKLFLATAISLGVSSAVLAADYDLKFGMNAGTSSNEYKAAEMFAKEVKEKSQGKIEISLYPSSQLGDDRAMLKQLKDGSLDFTFAESARFQLFYPEAAVFALPYVISNYNVAQKALFDTEFGKDLIKKMDKDLGVTLLSQAYNGTRQTTSNRAINSIADMKGLKLRVPNAATNLAYAKYVGASPTPMAFSEVYLALQTNAVDGQENPLAAVQAQKFYEVQKFLAMTNHILNDQLYLVSNETYKELPEDLQKVVKDAAENAAKYHTKLFVDGEKDLVTFFEKQGVKITHPDLVPFKESMKPYYAEFVKQTGQKGESALKQIEAINP |
